# Human Tacheng Tick Virus 2 Infection, China, 2019

**DOI:** 10.3201/eid2702.191486

**Published:** 2021-02

**Authors:** Zhihui Dong, Meihua Yang, Zedong Wang, Shuo Zhao, Songsong Xie, Yicheng Yang, Gang Liu, Shanshan Zhao, Jing Xie, Quan Liu, Yuanzhi Wang

**Affiliations:** Shihezi University, Shihezi, China (Z. Dong, M. Yang, Shuo Zhao, Y. Yang, G. Liu, Shanshan Zhao, Y. Wang);; Foshan University, Foshan, China (Z. Wang, Q. Liu);; First Affiliated Hospital of Shihezi University, Shihezi (S. Xie, J. Xie);; Shihezi People’s Hospital, Shihezi (Y. Yang)

**Keywords:** tickborne diseases, vector-borne infections, zoonoses, parasites, viruses, Tacheng tick virus, ticks, phleboviruses, China

## Abstract

We used metagenomic analysis to identify Tacheng tick virus 2 infection in a patient with a history of tick bite in northwestern China. We confirmed the virus with reverse transcription-PCR, virus isolation, and genomic analysis. We detected viral RNA in 9.6% of ticks collected from the same region.

Emerging pathogenic tickborne viruses have attracted much attention because of the increasing incidence of tickborne viral diseases and their effects on human health (*1*–*4*). In 2015, high-throughput sequencing of samples from ticks in China revealed several novel phleboviruses, including Tacheng tick virus 2 (TcTV-2), Changping tick virus 1, Bole tick virus 1 (BlTV-1), Lihan tick virus, Yongjia tick virus 1, and Dabieshan tick virus (*5*). However, the risk for human infection from these viruses is not yet known. We report on TcTV-2 infection in patient in China and describe methods for virus isolation and genomic analysis.

## The Study

The patient was a 38-year-old man who lived in northwestern China and had frequent contact with horses and sheep. On May 29, 2019, he noticed a tick embedded on his left upper arm and removed it himself. He noted a localized rash with slight pain and discomfort. On June 16, fever developed and soon after the patient had chills, severe fatigue, headache, anorexia, nausea, and vomiting. On June 20, he was admitted to the local hospital with a temperature of 37.9°C, which increased to 39.5°C the next day. The patient was initially given intravenous cefotaxime sodium and levofloxacin for 3 days for suspected tickborne bacterial disease, but these treatments did not alleviate his symptoms. 

**Figure 1:**
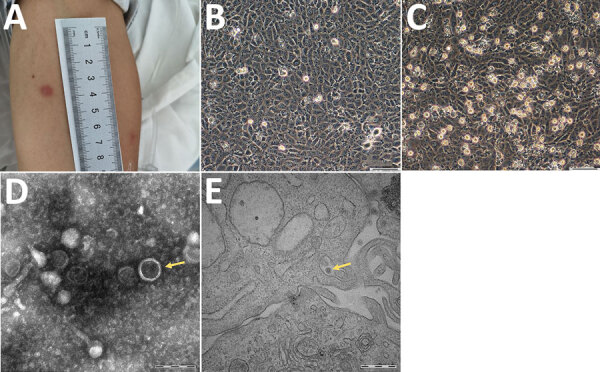
Clinical and morphological features of Tacheng tick virus 2 in a patient, China. A) Erythema at the site of tick bite on the anterior surface of the patient’s left arm. B) Human hepatocellular carcinoma (SMMC-7721) cells without TcTV-2 infection; magnification × 100. Scale bar indicates 50 μm. C) TcTV-2–infected SMMC-7721 cells showing cytopathic effects visible by light microscopy; magnification × 100. Scale bar indicates 50 μm. D) Negatively stained virions purified from TcTV-2–infected SMMC-7721 cells (arrows); magnification × 25,000. Scale bar indicates 200 nm. E) Transmission electron microscopy image of TcTV-2–infected SMMC-7721 cells (arrows); magnification × 50,000. Scale bar indicates 500 nm. TcTV-2, Tacheng tick virus 2.

On June 24, the patient was admitted to the First Affiliated Hospital of Medical College of Shihezi University in Shihezi. Physical examination showed erythema at the bite site (Figure 1, panel A) and neck stiffness. Cerebrospinal fluid (CSF) analysis showed a total of 1.07 × 10^8^ nucleated cells (92% hyaline leukocytes and 8% pleocaryocytes), an increased protein level (0.99 g/L), and decreased levels of CSF glucose (2.3 mmol/L) and chloridion (116.0 mmol/L). The patient was given intravenous ceftriaxone for 12 days, but still experienced headache, nausea, and vomiting, and his erythema was not decreasing.

Blood, throat swabs, urine, and CSF samples were obtained from the patient on days 9, 16, and 40 after illness onset. We tested the patient samples by PCR or reverse transcription-PCR (RT-PCR) for potential tickborne pathogens, including severe fever with thrombocytopenia syndrome virus, tickborne encephalitis virus, *Borrelia burgdorferi* sensu lato, *Anaplasma*, *Babesia*, *Rickettsia* spp., Tacheng tick virus 1, TcTV-2, Tacheng tick virus 5, BlTV-1, and Bole tick virus 4 (*2*). We detected TcTV-2 by metagenomic analysis on blood collected on day 9 and confirmed the virus by RT-PCR targeting the large (L) gene (Appendix Tables 1, 2). We detected TcTV-2 in blood, throat swabs, and urine samples from the patient. We ruled out bacterial infection in blood and CSF by using routine culture methods and 16S rRNA gene broad-range PCR, which confirmed that no bacterial infection occurred in this patient. 

On July 18, the patient was admitted to the hospital again. He was given intravenous acyclovir for 12 days and his clinical symptoms and erythema vanished without any sequelae (Appendix Table 3).

To isolate the virus, we inoculated human hepatocellular carcinoma (SMMC-7721) cells, African green monkey kidney (Vero) cells, baby hamster kidney cells, and human foreskin fibroblasts with the serum samples collected during early illness onset (Appendix Figure 2). We performed electron microscopy analysis on infected cells showing cytopathic effect, as described previously (*6*). After incubation, only the SMMC-7721 cells demonstrated cytopathic effect associated with TcTV-2 after several passages (Figure 1, panels B,C; Appendix Figure 3). The virions were spherical with a diameter of ≈90–100 nm (Figure 1, panel D). The virions could be seen in the cytoplasm of infected SMMC-7721 cells on transmission electron microscopy (Figure 1, panel E). We tested for TcTV-2–specific antibodies by using immunofluorescence assay. Serologic detection showed that TcTV-2 IgM titer in serum samples decreased from 1:40 on day 9 to 1:10 on day 40 after illness onset, and IgG titer increased from 1:10 on day 9 to 1:80 on day 40 (Table). 

**Table:** Results of immunofluorescence assay in detection of Tacheng tick virus 2 infection in a human, China*

Days after illness onset	Sample type	IFA titer	
		IgM	IgG
Day 9	Serum	1:40	<1:10
	Urine	<1:10	<1:10
	CSF	<1:10	<1:10
Day 16	Serum	1:20	1:10
	Urine	<1:10	<1:10
	CSF	<1:10	<1:10
Day 40	Serum	<1:10	1:80
	Urine	<1:10	<1:10
	CSF	<1:10	<1:10

*CSF, cerebrospinal fluid; IFA, immunofluorescence assay.

We isolated total RNA from infected cells and used the isolates to amplify the L and small (S) gene segment sequences by using primers based on our metagenomic analysis (Appendix Table 2, 4). The obtained L segment of TcTV-2 from the patient (GenBank accession no. MN567189) showed 98.8% (6,579/6,659) identity to the L segment of strain TC252 (GenBank accession no. KM817684) and the S segment from the isolate (GenBank accession no. MN567190) showed 99.2% (2,169/2,185) identity to the S of strain TC252 (GenBank accession no. KM817744).

**Figure 2:**
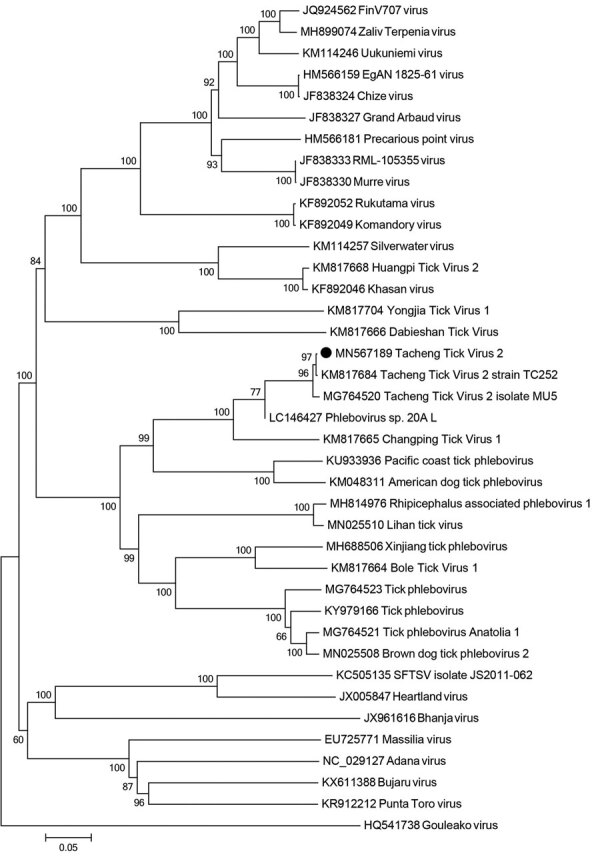
Phylogenetic analysis based on partial amino acid sequences of the L segment of tickborne viruses. Black dot indicates Tacheng tick virus 2 isolated from the patient in this study. The tree is constructed by using the neighbor-joining method in MEGA version 7.0 (https://www.megasoftware.net) and tested by the bootstrap method with 1,000 replications. Scale bar indicates nucleotide substitutions per site.

Phylogenetic analysis suggested that TcTV-2, together with Phlebovirus sp. 20A L, Pacific coast tick phlebovirus, Changping tick virus 1, BlTV-1, Lihan tick virus, Yongjia tick virus 1, Dabieshan tick virus, American dog tick phlebovirus, *Rhipicephalus*-associated phlebovirus 1, Xinjiang tick phlebovirus, tick phlebovirus, and brown dog tick phlebovirus 2 formed a separate branch (Figure 2; Appendix Figure 1). An M segment has yet to be detected in any of these viruses (*5*,*7*–*12*). 

To identify local natural virus hosts in the environment, 345 adult ticks were collected in the area where the patient lived, including 108 *Dermacentor marginatus*, 183 *D*. *nuttalli*, 12 *D*. *silvarum*, and 42 *Hyalomma asiaticum*. We extracted total RNA of each tick and detected TcTV-2 by using RT-PCR with TcTV-2–specific primers (Appendix Table 1). Among 345 ticks, 33 (9.6%) carried TcTV-2. We noted high infection rates in *D. silvarum* (16.7%), *D*. *marginatus* (14.8%), *H. asiaticum* (11.9%), and *D. nuttalli* (5.5%). We obtained the partial fragments of the S segment of TcTV-2 in ticks and phylogenetic analyses showed that sequences from TcTv-2 in ticks were closely related to the isolate from the patient (Appendix Figure 1, Table 5).

We tried to obtain the medium (M) segment of TcTV-2 by designing a set of primers based on the conservative sequences of M segments from 15 typical phleboviruses (Appendix Table 6). We used these primers to amplify the M segment from both the patient and positive ticks detected by sequencing the L and S segments by using RT-PCR. We further analyzed the metagenomic sequences, but the results were negative.

## Conclusions

Among currently known emerging tickborne phleboviruses, severe fever with thrombocytopenia syndrome virus and Heartland virus have been reported to infect humans and cause multiple organ damage, including to the liver and kidneys (*1*,*13*). In this study, TcTV-2 did not show any growth in Vero, human foreskin fibroblasts, or baby hamster kidney 21 cells, but had low level replication and growth in SMMC-7721 cells, indicating that the virus is not well adapted to mammals and likely is more common in arthropods than in mammals.

Transmission electron microscopy showed that TcTV-2 might harbor glycoprotein encoded by the M gene segment. The lack of M sequence data on homology-based approaches could indicate that insufficient homology exists between these viruses to detect the M gene in this manner. Sequencing methods that obtain a greater depth of coverage might help obtain the missing M sequences. To increase the virus titer and the likelihood of obtaining the M sequence, we recommend performing deep sequencing on the isolated virus.

TcTV-2 previously was identified in *D*. *marginatus* ticks from China (*5*) and in *H. marginatum* ticks from Turkey (*12*). We detected TcTV-2 in *D*. *nuttalli*, *D*. *silvarum, H*. *asiaticum* ticks and in blood, urine, and throat swab samples from a patient with febrile illness. Our findings suggest that person-to-person transmission might be possible through direct contact with body fluids or by droplet transmission. In addition, we noted more tick species found in northwest China that could act as TcTV-2 vectors (*14*), but this finding should be verified in further studies. Nonetheless, our study demonstrates that TcTV-2 could be emerging and infecting humans. Clinicians should consider TcTV-2 infections in patients with febrile illness and recent history of tick bites.

Appendix Additional information on Tacheng tick virus 2 isolated from a patient in China.
