# Supplementary material for: Human Tacheng Tick Virus 2 Infection, China, 2019
Source: Emerg Infect Dis. 2021 Feb;27(2):594–8. doi: 10.3201/eid2702.191486 (PMC7853585; doi:10.3201/eid2702.191486)
Supplement: Appendix — Additional information on Tacheng tick virus 2 isolated from a patient in China. [file 19-1486-Techapp-s1.pdf]

Article DOI: https://doi.org/10.3201/eid3201/191486

Human Tacheng Tick Virus 2 Infection, China, 2019

Appendix

Appendix Table 1. Primer sequences of nested reverse transcription-PCR for detection of multiple tickborne pathogens in a study of Tacheng tick virus 2 in a patient, China, 2019

Targeting pathogens	Primer name	Primer sequence (5′→3′)	Gene segment	Product size, bp
Tacheng tick virus 2	F1	ATCTCCTCAACGGCAACTAT	359–812	454
	R1	GACATGCGGTTCTTCATTTT		
	F2	TCAACGGCAACTATGAGGAT	365–616	252
	R2	CTGGCTTGTATTGGAAGGAC		
Tacheng tick virus 5	F1	TGGTCAGATGGGAGGAAT	2105–2709	605
	R1	CCGCCCTAGCAAAGAAAT		
	F2	TATGGTTCCTCCAAAAGC	2243–2709	467
	R2	CCGCCCTAGCAAAGAAAT		
Bole tick virus 1	F1	GCAARCCTGGAAGTCAAGCC	4963–5754	812
	R1	AGAGCACCCGCAGCATAGTC		
	F2	GCAARCCTGGAAGTCAAGCC	4963–5703	741
	R2	CTCTGAGTGGGTTCCTGATT		
Bole tick virus 4	F1	ACGCTTGTAGGGCTCAGGTGTC	517–1079	563
	R1	CTGGTCGKCAATCTGRATGCAACA		
	F2	CTCAGGTGTCCTTTGACGATTAT	529–1079	551
	R2	CTGGTCGKCAATCTGRATGCAACA		

Appendix Table 2. Sequences of large gene contigs of Tacheng tick virus 2 in patient by metagenomic analysis and their identities to the reference strain*

Contig	Location	Sequence (5′→3′)	Sequence identity, no./No. (%)
1	252–372	TCCTCACCCTTCAAGCCTCAACTCACTTTTTCCATGATTTCACGTTCCAGTACC<br>TGTCACGAGACACAGATGTTCCCTTCAAGAAATTCTATGAGACTGTGAATGAT<br>GGCTTTGACAACCACACCCCTGATGTCATAATTGAAGACATCCAAGG	152/153 (99)
2	921–1101	GGATCTCAATTGGCTAGAATGCACCAAGAAGGTAAATGAGTACAGGACTTCCT<br>TCCACAAGCCTCGCAAAATGCGAGGGAGGCTTTCACATAAATCAACTATCCC<br>CTTCCCAGGCTTTATGCCCATGGTATCTGAATATCCTTCCATTAGCGTCAAGG<br>AGATCCTCAAAAAACCCTCCAACTTCCCCCA	186/187 (99)
3	1668–1788	GGGGCCGGGTGAGTACATTATCAAGAAGCTGAGGAACTTTGAGGTCTTCCTG<br>CTCATAAAGCCAACCAAGAGCAATGGGCCGATGTTCGTATCTCTGGCTTGGC<br>ATGCAAAAGATGTGTCTAACACATACCTTACAGACTACAT	135/141 (96)
4	1708–1828	GTCTTCCTGCTCATAAAGCCAACCAAGAGCAATGGGCCGATGTTCGTATCTCT<br>GGCTTGGCATGCAAAAGATGTGTCCAACACATACCTTACAGACTACATGGTGT<br>TCAAGGCCACTCAGCAGGTGGGAGACTGGTGCTGGACTGAATTCCATTCCTT<br>AAAACCATCCA	163/169 (96)
5	2088–2208	CGGGCTTTGTGTCAGATCCCTGTCTGCCCCATCCTCACAAGATGATCGAAAA<br>GCTTCCAG<br>ATGTGGCCAGGACCAAACTCCAGGTCTGGCTGATCAACAAGAGCCTCAACCT<br>CATGCAAG<br>ACATTGCTCTGAGGCCTTATCAGCCA	143/145 (99)
6	2238–2558	CCGCAGAATGCATTCAATACATCCATGTGCATTGATTGCACTATGTTGGGGCC<br>CCAGGTTGCTTTGAGGTGCACCAGTGCCACGTCACACACGTACTTCTGAAAT<br>GACACGGAGTACTCATGTGTCTTTATGTCGTCCAGAGGAGGATCCTTCCGGC<br>CTA	159/160 (99)
7	4547–4667	CCACATATATGTCCAAAGAGGGTCAAAACGTGCAGGGTCGAAGGAAGACAGT<br>CCAAACCAGGGTGGTGGTTTTCCACAGAGAAGAGGCAATGCGAGCAAGGCC<br>AGAAGATGTCCTCACAGATGTATGGTGGGGTCGA	134/135 (99)
8	4677–4857	GGCGGGCCTCAGAAGGAGTGGCCTCACTTTGCCTGCACTGAAGGAGCATTTT<br>GAGCAACTGCAGAGAGTGCTCCCATGGCTAACCAAGGATCCCAATGAGTCAT<br>TGAGAAGATCCCCTTTCTTGCACCACCATCAACTCAGGAACTTTCTCTCGCGG<br>ATGGAGATCCAAGGTAGAGAAGTCAGGCT	179/183 (98)

*GenBank accession number of the reference strain is KM817684.

Appendix Table 2. Sequences of N gene contigs of Tacheng tick virus 2 isolated in a patient by metagenomic analysis and their identities to the reference strain*

Contig	Location	Sequence (5′→3′)	Sequence identity, no./No. (%)
1	752–872	CCATGATTGTGCTCTACCTCACAAGGGGCACAAACACAGAAAAAATGAAGAACCGC<br>ATGTCCGAGATGGGCAAGATGCTGATGGATCGGCTGGAAAAGCAGTACCAGATCC<br>GGAAGGGTGCCGTGGCGCCCAAGGAGATCACCCTGGCCAGGGTTGCCCTCACCT<br>ACCCGG	170/171 (99)
2	774–894	AAGGGGCACAAACACAGAAAAAATGAAGAACCGCATGTCCGAGATGGGCAAGATG<br>CTGAT<br>GGATCGGCTGGAAAAGCAGTACCAGATCCGGAAGGGTGCCGTGGCGCCCAAGGA<br>GATCAC<br>CCTGGCCAGGGTTGCCCTCACCTACCCGG	148/149 (99)

*GenBank accession number of the reference strain is KM81744.

Appendix Table 3. Diagnostic and treatment information for patient with Tacheng tick virus 2 infection, China, 2019*

Days post illness onset	Day 9				Day 16			Day 40			
Sample type	Blood	Urine	Throat swab	CSF	Blood	Urine	Throat swab	Blood	Urine	Throat swab	CSF
Nested RT-PCR	+	+	+	–	+	+	+	+	+	+	–
Virus isolation	+	NA	NA	NA	NA	NA	NA	NA	NA	NA	NA
Co-infection		–				–			–		

Laboratory findings, date	Jun 18	Jun 25	Jun 28	Jul 4	Jul 18	Jul 29
Blood						
Leukocyte count ($\times 10^9$/L)	11.62	10.9	NA	NA	5	NA
Lymphocyte count ($\times 10^9$/L)	0.63	1.90	NA	NA	1.6	NA
Monocyte count ($\times 10^9$/L)	0.16	1.4	NA	NA	0.7	NA
Neutrophil count ($\times 10^9$/L)	10.82	7.51	NA	NA	2.31	NA
Platelet count ($\times 10^9$/L)	267	280	NA	NA	211	NA
Red cell count ($\times 10^9$/L)	4.79	4.91	NA	NA	4.75	NA
Hypersensitive C-reactive protein (mg/L)	2.0	0.5	NA	NA	0.87	NA
ALT (U/L)	NA	10.1	12.7	NA	16.9	NA
AST (U/L)	NA	13.8	16.8	NA	16.8	NA
Albumin (g/L)	NA	40.9	40.8	NA	40.8	NA
Globin (g/L)	NA	26.7	24.8	NA	26.7	NA
Potassium (mmol/L)	NA	3.63	3.82	NA	3.82	NA
Sodium (mmol/L)	NA	139.0	139.00	NA	139	NA
Chloride (mmol/L	NA	101.00	108.00	NA	108.00	NA
Calcium (mmol/L)	NA	2.14	2.17	NA	2.14	NA
Fibrinogen (g/L)	NA	NA	3.29	NA	1.34	NA

Days post illness onset	Day 9				Day 16			Day 40			
			Throat				Throat			Throat	
Sample type	Blood	Urine	swab	CSF	Blood	Urine	swab	Blood	Urine	swab	CSF
D-dimer (ng/ml)	NA		8.03		NA		NA	NA		NA	
Prothrombin activity (%)	NA		NA		73.40		NA	68.50		NA	
Cerebrospinal fluid											
Pressure	NA		195		130		125	130		140	
Appearance	NA		achromatic		achromatic		achromatic	achromatic		achromatic	
Transparency	NA		transparent		transparent		transparent	transparent		transparent	
Leukocyte count (×10^6/L)	NA		107		34		32	19		32	
Hyaline leukocyte	NA		92%		NA		NA	NA		NA	
Pleocaryocyte	NA		8%		NA		NA	NA		NA	
Glucose	NA		2.30		2.82		2.85	2.20		3.17	
Chloridion	NA		116.0		122.0		124.0	122.0		126	
Protein quantification	NA		988.50		654		654.00	475		414	

Clinical treatment	
Days 5–8 after illness onset	Antodin (2 mL intramuscularly × 1)
	Cefotaxime Sodium (3 g intravenously 2×/day on days 5 and 6)
	Levofloxacin (0.4 g intravenously 1×/day on day 8)
Day 9–21 after illness onset	Ceftriaxone sodium (1 g intravenously 2×/day on days 9–21)
	Invert sugarand electrolytes injection (500 mL given intravenously 2×/day on days 9–21)
	20% Mannitol injection (250 mL intravenously 2×/day on days 9 and 10)
	20% Mannitol injection (125 mL intravenously 3×/day on day 11)
	Esmerazole sodium (40 mg does intravenously 4×/day × on days 9 and 10)
	Aluminum phosphate gel (20 g intravenously 2×/day on days 9 and 10)
	20% Mannitol injection (125 mL intravenously 3×/day on days 15–17)
Day 33–45 after illness onset	Aciclovir (0.25 g intravenously 3×/day on days 33–45)
	Lansoprazole (30 mg intravenously 1×/day on days 33–43)
	Ringer sodium lactate (500 mL intravenously 1×/day on days 33–42)

*ALT, alanine aminotransferase; AST, aspartate aminotransferase; CSF, cerebrospinal fluid; NA, not available because test was not performed or results were not reported; RT-PCR, reverse transcription-polymerase chain reaction; +, positive; –, negative.

Appendix Table 4. Primer sequences used to amplify the complete genome of Tacheng tick virus 2 isolated from a patient, China

Genome segment	Forward (location) (5′→ 3′)	Reverse (location) (5′→3′)
Large segment	LF1: AAGGGCACGCCACAACCC	LR511: GTTGGTCGTTGCCTTCCACG
	LF253: CTCACCCTTCAAGCCTCAACTC	LR992: TATGTGAAAGCCTCCCTCGCA
	LF812: CGGACCAGGACTACATCAAGAAG	LR1930: ATCCATCTGCTCCCTCCAACC
	LF1824: TCAGCAGGTGGGAGATTGGT	LR2743: ACACTGACGCAAGATGTGGTG
	LF2721: CACGCCCATGAACACACAT	LR3787: AACCACCTCCCTCAAGGACACT
	LF3725: CAATAGTGGCGAGGCAGGA	LR4624: GCTCGCATTGCCTCTTCTCT
	LF4376: GCACTTCTTGGATGGCACCT	LR5613: TTCTGCTGTCGTCCTCGTTG
	LF5289: GGGCACTGTCAACGGTCAAT	LR6546: GGCCCAACATCGTAATCCTCT
	LF6245: TAGAACAAAAGGAAGGCTTCTTCGAC	LR6632: CATAATCTCAAAGACCCTATACTGCCAC
Small segment	SF1: TGCCCCCCTCACTAAACAC	SR543: CTGGTGTTGATGCGTTGTCC
	SF344: CCTGCGTGGAAATTGATCTC	SR1597: ATCAGACGCACATCCATTCG
	SF1099: CTGGCAGAGTTCTCCAAGGTCA	SR1840: TTCCCTTGTGCTGCCATCCT
	SF1752: TAGCATTATGAGTGATGCCCAA	SR2185: TTCATTTAGTTTTACCTAGCTCCGAC

Appendix Table 5. Detection of Tacheng tick virus 2 by reverse transcription PCR in ticks collected from 9 counties and cities, Xinjiang Province, China

Tick species	County of sampling collection	Host	No. sampled	No. positive (%)
Dermacentor nuttalli	Qinghe	Sheep	86	7 (8.1)
	Wenquan	Free*	77	2 (2.6)
	Wusu	Free*	20	1 (5.0)
D. silvarum	Jinghe	Long-tailed ground squirrel	12	2 (16.7)
D. marginatus	Fuyun	Horse	44	10 (22.7)
	Gongliu	Sheep	32	2 (6.3)
	Xinyuan	Sheep	32	4 (12.5)
Hyalomma asiaticum	Shawan	Free*	21	1 (4.8)
	Fuhai	Camel	21	4 (19.0)
Total			345	33 (9.6)

*Ticks collected from the sampling the environment.

Appendix Table 6. Primers used in a study of Tacheng tick virus 2 isolated from a human patient, China*

Primer name	Primer sequence (5′→3′)	Purification method
FinV707 virus M segment FW-1801	GATGTCATCTCATGGGAGCTTG	PAGE
FinV707 virus M segment DW-2240	CTCTTATTACAGACTGTATGGAA	PAGE
FinV707 virus M segment DW-2290	GTCGGCCAGAACGTGACTGAACC	PAGE
Zaliv virus M segment FW-1801	GGTGCCATTTGATGGGAGCTTG	PAGE
Zaliv virus M segment DW-2240	CTTCTATTGCAGACTGTATGGAA	PAGE
Zaliv virus M segment DW-2290	GTTGGCCAGAATGTGACAGAACC	PAGE
Uukuniemi virus M segment FW-1801	GATGCCATCTCATGGGAGCCTG	PAGE

Primer name	Primer sequence (5′→3′)	Purification method
Uukuniemi virus M segment DW-2240	CTTCTATTGCAGACAGTATGGAA	PAGE
Uukuniemi virus M segment DW-2290	GTTGGCCAGAAAGTGACTGAACC	PAGE
Chize-EgAN virus M segment-FW 1801	GGAGGTGCCATCTAATGGGAG	PAGE
Chize-EgAN virus M segment DW-2240	CTTCGATTGCAAACAGTATGGAA	PAGE
Chize-EgAN virus M segment DW-2290	CCAGAAGGTAACTGAGCCCAC	PAGE
Murre-RML-Precarious virus M segment FW-2040	TCTGAGCATTCAAACATGTTGTA	PAGE
Murre-RML virus M segment DW-2400	GATGGGTCAATAATGCTAGAT	PAGE
Precarious virus M segment DW-2400	GAAGGGTCAATGATGCTAGAT	PAGE
Murre-RML virus M segment DW-2610	CCCGGTTCACAATTATAACA	PAGE
Precarious virus M segment DW-2610	CCCGGTTCACAGTTATAACA	PAGE
Gissar-Grand arbaud virus M segment FW-270	GGCAGAAATGGATTAATCATGATGG	PAGE
Gissar-Grand arbaud virus M segment FW-550	AACTGGTTTTGGATTGATGG	PAGE
Gissar-Grand arbaud virus M segment DW-960	GAWCCTAGACATGCCCTGGTA	PAGE
Komandory-Rukutama virus M segment FW-30	ATGGAATCAACTATGAGAGGG	PAGE
Komandory-Rukutama virus M segment FW-1860	CTCGGAACAAGAAGATGCCATCTC	PAGE
Komandory-Rukutama virus M segment FW-2070	GTGTTTAACATGTTTGAATGT	PAGE
Komandory-Rukutama virus M segment DW-2230	CTCAGATTCTCCAAAGCATCTGTC	PAGE
Komandory-Rukutama virus M segment DW-2390	ATCATCAATATGGACTTTTGC	PAGE
Huangpi-Khasan virus M segment FW-1790	TGTCTAAAGAGTGATCTTTACTGG	PAGE
Khasan-Silverwater virus M segment FW-1720	TGTTGACTAGCCAGWCTGGTGA	PAGE
Khasan-Silverwater virus M segment DW-2310	CAYTGTATTTCTCCAAGCTT	PAGE
Huangpi-Khasan virus M segment DW-2260	TGCATGGTGCATGGAACATCTC	PAGE
Khasan-Huangpi Tick virus M segment FW-290	ATGGACTGTTCTGGTGGTAGG	PAGE
Silverwater virus M segment FW-280	GTGAGATGGACTGCTCTGGTGG	PAGE
Khasan-Huangpi Tick virus M segment FW-450	GATGACATGATCTGTCAGTTTGAG	PAGE
Silverwater virus M segment FW-450	GATGACATGATCTGCCAGTTCGAG	PAGE
Khasan-Huangpi Tick virus M segment DW-720	GAGCATCTCCTGTACATCTTCCTG	PAGE
Silverwater virus M segment DW-720	CCAGTGCATTTCCCAGCCTTACA	PAGE

*We designed and synthesized a set of primers based on 15 tri-segment phleboviruses. In brief, we identified 14 tri-segment phleboviruses that cluster with Tacheng tick virus 2 and its related viruses based on phylogenetic analysis of the amino acid sequence for large segments by the neighbor-joining method. Viruses included Rukutama virus, Precarious point virus, RML-105355 virus, Murre virus, Komandory virus, Grand Arbaud virus, Silverwater virus, Huangpi tick virus 2, Khasan virus, Uukuniemi virus, Zaliv Terpenia virus, FinV707 virus, Chize virus, EgAN 1825–61 virus (Figure 2). We did not use Gissar virus because of its short sequence in GenBank. We then found common sequences through nucleotide sequence alignment function of 6 groups based on 15 trisegment phleboviruses by using DNAMAN software (Lynnon Biosoft Bioinformatic Solutions, https://www.lynnon.com). Finally, we used the selected common sequences to design primers. To enhance efficacy and possibility of reverse transcription PCR, we designed sets of seminested PCR primers for each virus group. M, medium.

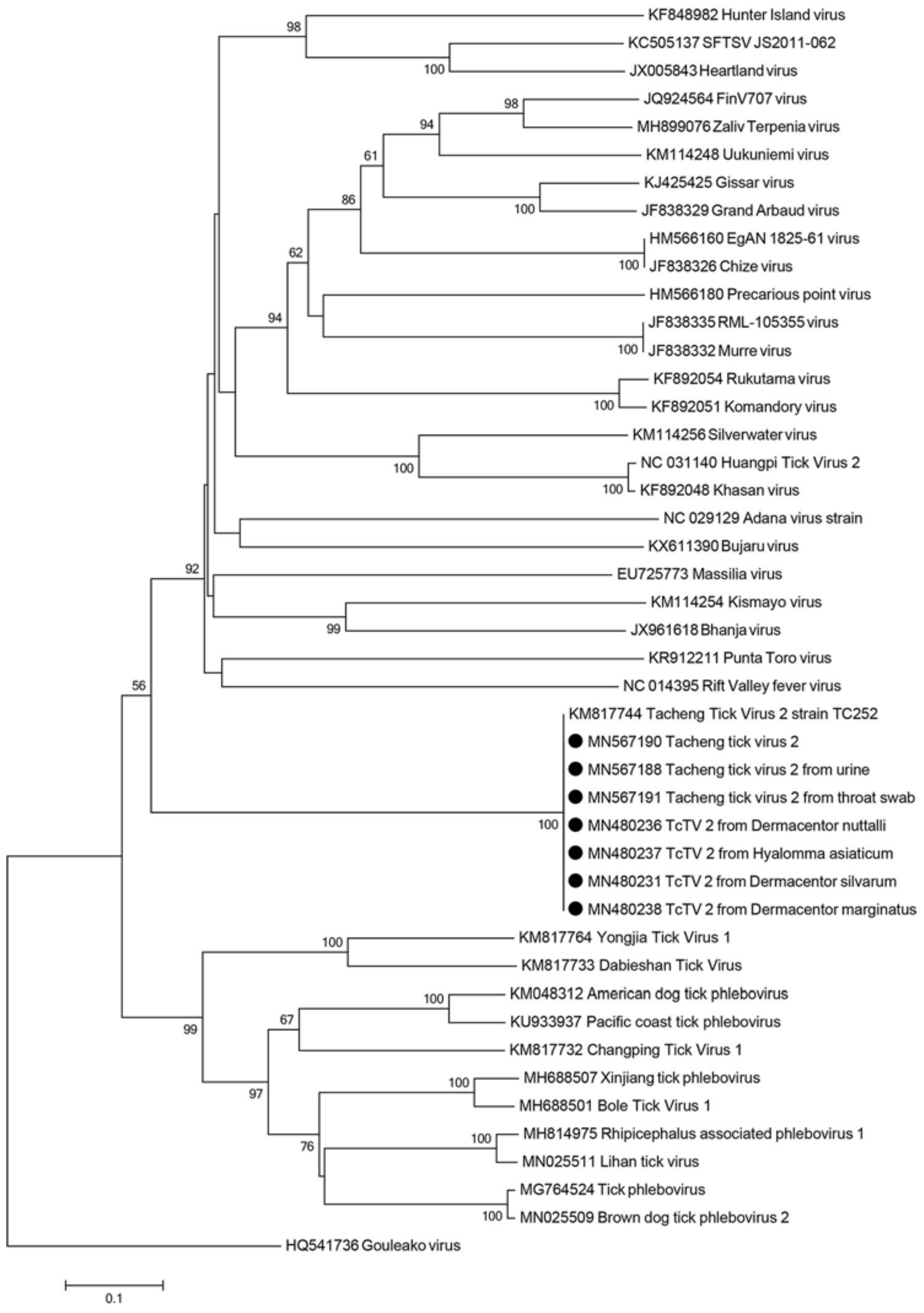

Appendix Figure 1. Phylogenetic analysis based on partial amino acid sequences of the small segment of tickborne viruses. The tree was constructed using the neighbor-joining method by using MEGA version 7.0 (https://www.megasoftware.net). Black dots indicate Tacheng tick virus 2 isolated in this study (GenBank accession nos. MN567188, MN567190, MN567191, MN480231, MN480236, MN480237, and MN480238). Scale indicates amino acid substitutions per site.

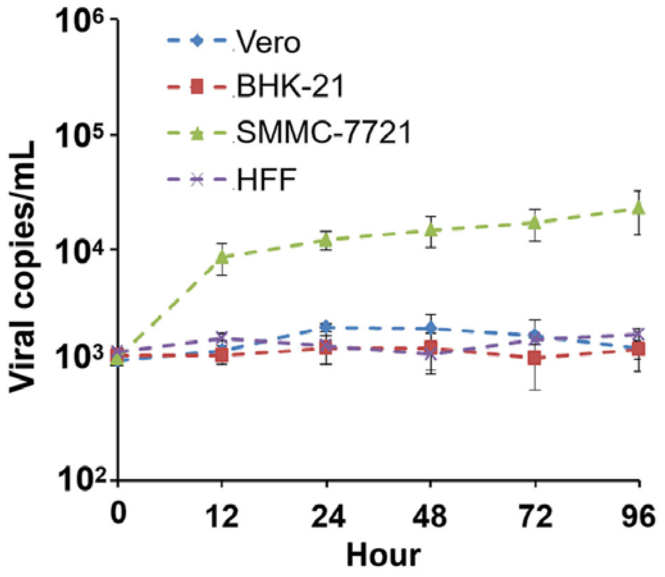

Appendix Figure 2. Viral copies of Tacheng tick virus 2 over time in human hepatocellular carcinoma (SMMC-7721), African green monkey kidney (Vero), hamster kidney (BHK-21), and human foreskin fibroblast (HFF) cells. Estimates from the 2 technical replicates were averaged and error bars indicate standard deviation (SD) across biological replicates.

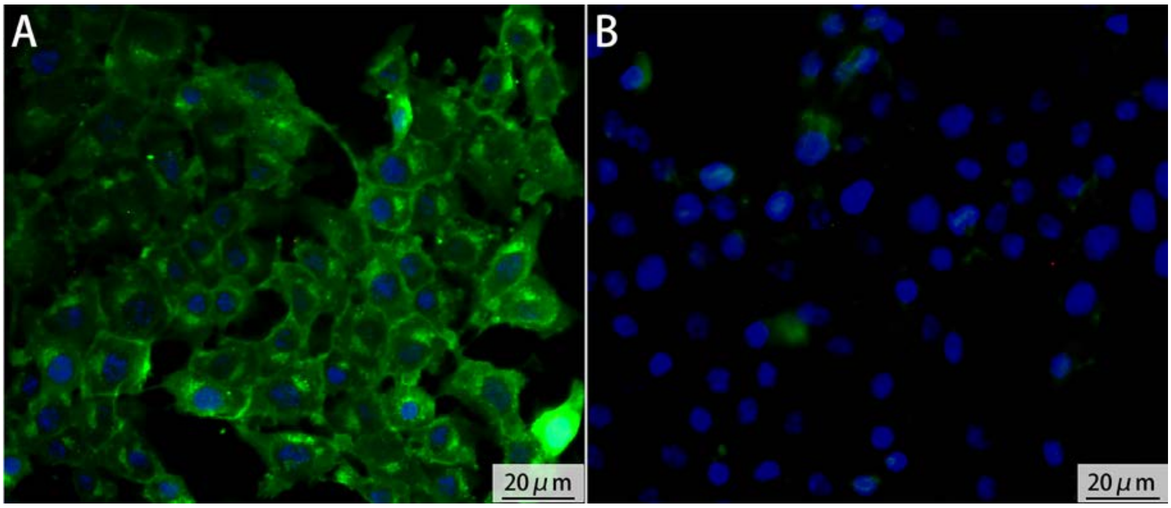

Appendix Figure 3. The detection of human hepatocellular carcinoma (SMMC-7721) cells infected with Tacheng tick virus 2 by immunofluorescence assay. A) The virus grown in SMMC-7721 cells detected by IFA; magnification × 200. B) SMMC-7721 cells without TcTV-2 infection, stained by the patient's convalescent serum; magnification × 200. IFA, immunofluorescence assay; TcTV-2, Tacheng tick virus 2.
